# 5-Cyclo­hexyl-3-ethyl­sulfinyl-2-(3-fluoro­phen­yl)-1-benzo­furan

**DOI:** 10.1107/S1600536813023180

**Published:** 2013-08-23

**Authors:** Hong Dae Choi, Pil Ja Seo, Uk Lee

**Affiliations:** a Department of Chemistry, Dongeui University, San 24 Kaya-dong, Busanjin-gu, Busan 614-714, Republic of Korea; b Department of Chemistry, Pukyong National University, 599-1 Daeyeon 3-dong, Nam-gu, Busan 608-737, Republic of Korea

## Abstract

In the title compound, C_22_H_23_FO_2_S, the cyclo­hexyl ring adopts a chair conformation. The dihedral angle between the mean plane [r.m.s. deviation = 0.013 (2) Å] of the benzo­furan ring system and the mean plane [r.m.s. deviation = 0.009 (2) Å] of the 3-fluoro­phenyl ring is 24.80 (4)°. In the crystal, mol­ecules are connected by C—H⋯O hydrogen bonds, forming chains along [10-1]. These chains are linked *via* C—H⋯F hydrogen bonds, forming a three-dimensional structure. There are also inter­planar inter­actions present involving the furan ring of the benzo­furan ring system and the 3-fluoro­phenyl ring [centroid–centroid distance = 3.728 (2) Å].

## Related literature

For background information and the crystal structures of related compounds, see: Choi *et al.* (2011 ▶, 2012 ▶).
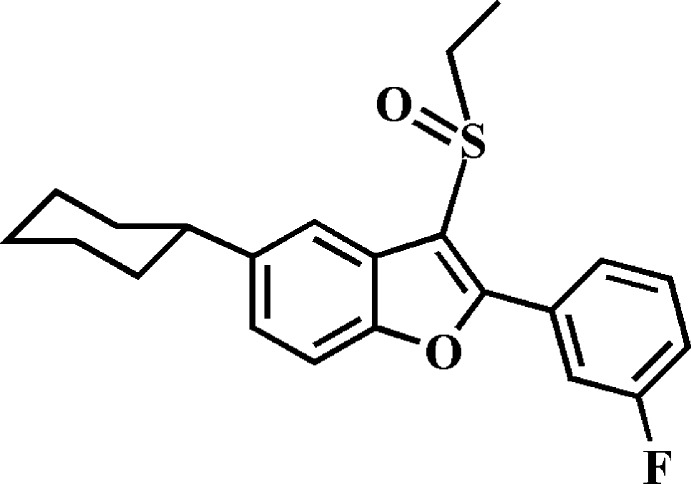


## Experimental

### 

#### Crystal data

C_22_H_23_FO_2_S
*M* *_r_* = 370.46
Monoclinic, 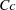

*a* = 15.1363 (5) Å
*b* = 15.9252 (6) Å
*c* = 10.6440 (6) Å
β = 133.053 (1)°
*V* = 1874.83 (14) Å^3^
*Z* = 4
Mo *K*α radiation
μ = 0.20 mm^−1^
*T* = 173 K
0.40 × 0.29 × 0.19 mm

#### Data collection

Bruker SMART APEXII CCD diffractometer
Absorption correction: multi-scan (*SADABS*; Bruker, 2009 ▶) *T*_min_ = 0.634, *T*_max_ = 0.746
8392 measured reflections
3085 independent reflections
2842 reflections with *I* > 2σ(*I*)
*R*_int_ = 0.030

#### Refinement

*R*[*F* ^2^ > 2σ(*F* ^2^)] = 0.032
*wR*(*F* ^2^) = 0.074
*S* = 1.04
3085 reflections
237 parameters
2 restraints
H-atom parameters constrained
Δρ_max_ = 0.17 e Å^−3^
Δρ_min_ = −0.22 e Å^−3^
Absolute structure: Flack (1983 ▶), 1045 (51%) Friedel pairs
Absolute structure parameter: 0.05 (7)

### 

Data collection: *APEX2* (Bruker, 2009 ▶); cell refinement: *SAINT* (Bruker, 2009 ▶); data reduction: *SAINT*; program(s) used to solve structure: *SHELXS97* (Sheldrick, 2008 ▶); program(s) used to refine structure: *SHELXL97* (Sheldrick, 2008 ▶); molecular graphics: *ORTEP-3 for Windows* (Farrugia, 2012 ▶) and *DIAMOND* (Brandenburg, 1998 ▶); software used to prepare material for publication: *SHELXL97*.

## Supplementary Material

Crystal structure: contains datablock(s) I. DOI: 10.1107/S1600536813023180/su2636sup1.cif

Structure factors: contains datablock(s) I. DOI: 10.1107/S1600536813023180/su2636Isup2.hkl

Supplementary material file. DOI: 10.1107/S1600536813023180/su2636Isup3.cml

Additional supplementary materials:  crystallographic information; 3D view; checkCIF report

## Figures and Tables

**Table 1:** Hydrogen-bond geometry (Å, °)

*D*—H⋯*A*	*D*—H	H⋯*A*	*D*⋯*A*	*D*—H⋯*A*
C16—H16⋯O2^i^	0.95	2.54	3.291 (3)	136
C3—H3⋯F1^ii^	0.95	2.54	3.438 (3)	159

Symmetry codes: (i) 
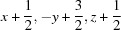
; (ii) 
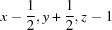
.

**Fig. 1.**
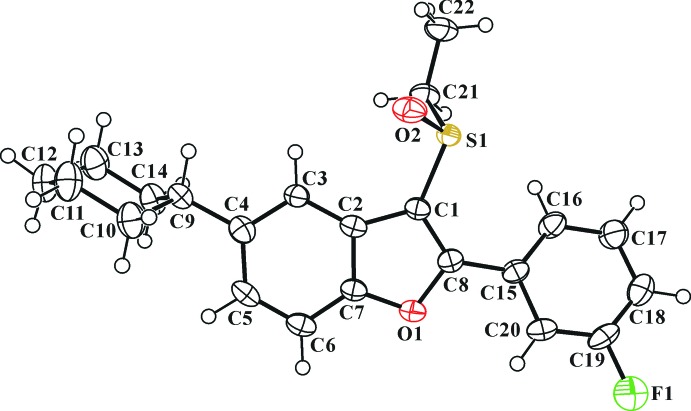
The molecular structure of the title molecule, with atom labelling. Displacement ellipsoids are drawn at the 50% probability level.

## supplementary crystallographic information

### 1. Comment

As a part of our continuing study of 5-cyclohexyl-1-benzofuran derivatives containing [2-(4-fluorophenyl)-3-methylsulfinyl] (Choi *et al.*, 2011) and [2-(3-fluorophenyl)-3-methylsulfinyl] (Choi *et al.*, 2012) substituents, we report herein on the crystal structure of the title compound.

The title compound crystallizes in the non-centrosymmetric space group *C*_c_ in spite of having no asymmetric C atoms.

In the title molecule, Fig. 1, the cyclohexyl ring adopts a chair conformation. The benzofuran ring system is essentially planar, with a mean deviation of 0.013 (2) Å from the mean plane defined by the nine non-H atoms. The 3-fluorophenyl ring is essentially planar, with a mean deviation of 0.009 (2) Å from the mean plane defined by the six non-H atoms. The dihedral angle formed by the benzofuran ring system and the 3-fluorophenyl ring is 24.80 (4)°.

In the crystal structure, molecules are connected by weak C—H···O hydrogen bonds (Table 1), forming chains along the [10-1] direction.

In the crystal, molecules are connected by C—H···O hydrogen bonds forming chains along the [10-1] direction (Table 1). These chains are linked via C-H···F hydrogen bonds forming a three-dimensional structure (Table 1). There are also inter-planar interactions present involving the furan ring of the benzofuran ring system and the 3-fluorophenyl ring [Cg1–Cg2^i^ = 3.728 (2) Å; Cg1 and Cg2 are the centroids of rings O1/C1/C2/C7/C8 and C15–C20, respectively; symmetry code: (i) x, -y+1, z-1/2].

### 2. Experimental

3-Chloroperoxybenzoic acid (77%, 202 mg, 0.9 mmol) was added in small portions to a stirred solution of 5-cyclohexyl-3-ethylsulfanyl-2-(3-fluorophenyl)-1-benzofuran (283 mg, 0.8 mmol) in dichloromethane (40 mL) at 273 K. After being stirred at room temperature for 5h, the mixture was washed with saturated sodium bicarbonate solution and the organic layer was separated, dried over magnesium sulfate, filtered and concentrated at reduced pressure. The residue was purified by column chromatography (hexane–ethyl acetate, 2:1 v/v) to afford the title compound as a colourless solid [yield 70%, m.p. 417–418 K; *R*_f_ = 0.68 (hexane-ethyl acetate, 2:1 v/v)]. Colourless block-like crystals, suitable for X-ray diffraction analysis, were prepared by slow evaporation of a solution of the title compound in acetone at room temperature.

### 3. Refinement

The reported Flack parameter was obtained using the TWIN/BASF procedure in SHELXL (Sheldrick, 2008). All H atoms were positioned geometrically and refined using a riding model: C—H = 0.95 Å for aryl, 1.00 Å for methine, 0.99 Å for methylene and 0.98 Å for methyl H atoms, with *U*_iso_(H) = 1.5*U*_eq_(C) for methyl H atoms and = 1.2*U*_eq_(C) for other H atoms. The positions of methyl hydrogens were optimized rotationally.

### Crystal data

**Table:**

C_22_H_23_FO_2_S	*F*(000) = 784
*M**_r_* = 370.46	*D*_x_ = 1.312 Mg m^−^^3^
Monoclinic, *C**c*	Melting point = 417–418 K
Hall symbol: C -2yc	Mo *K*α radiation, λ = 0.71073 Å
*a* = 15.1363 (5) Å	Cell parameters from 3291 reflections
*b* = 15.9252 (6) Å	θ = 2.2–27.2°
*c* = 10.6440 (6) Å	µ = 0.20 mm^−^^1^
β = 133.053 (1)°	*T* = 173 K
*V* = 1874.83 (14) Å^3^	Block, colourless
*Z* = 4	0.40 × 0.29 × 0.19 mm

### Data collection

**Table:**

Bruker SMART APEXII CCD diffractometer	3085 independent reflections
Radiation source: rotating anode	2842 reflections with *I* > 2σ(*I*)
Graphite multilayer monochromator	*R*_int_ = 0.030
Detector resolution: 10.0 pixels mm^-1^	θ_max_ = 27.0°, θ_min_ = 2.2°
φ and ω scans	*h* = −17→19
Absorption correction: multi-scan (*SADABS*; Bruker, 2009)	*k* = −20→20
*T*_min_ = 0.634, *T*_max_ = 0.746	*l* = −12→13
8392 measured reflections	

### Refinement

**Table:**

Refinement on *F*^2^	Secondary atom site location: difference Fourier map
Least-squares matrix: full	Hydrogen site location: difference Fourier map
*R*[*F*^2^ > 2σ(*F*^2^)] = 0.032	H-atom parameters constrained
*wR*(*F*^2^) = 0.074	*w* = 1/[σ^2^(*F*_o_^2^) + (0.0331*P*)^2^ + 0.634*P*] where *P* = (*F*_o_^2^ + 2*F*_c_^2^)/3
*S* = 1.04	(Δ/σ)_max_ = 0.001
3085 reflections	Δρ_max_ = 0.17 e Å^−^^3^
237 parameters	Δρ_min_ = −0.22 e Å^−^^3^
2 restraints	Absolute structure: Flack (1983), 1045 (51%) Friedel pairs
Primary atom site location: structure-invariant direct methods	Absolute structure parameter: 0.05 (7)

### Special details

**Table:**

Geometry. All esds (except the esd in the dihedral angle between two l.s. planes) are estimated using the full covariance matrix. The cell esds are taken into account individually in the estimation of esds in distances, angles and torsion angles; correlations between esds in cell parameters are only used when they are defined by crystal symmetry. An approximate (isotropic) treatment of cell esds is used for estimating esds involving l.s. planes.
Refinement. Refinement of F^2^ against ALL reflections. The weighted R-factor wR and goodness of fit S are based on F^2^, conventional R-factors R are based on F, with F set to zero for negative F^2^. The threshold expression of F^2^ > 2sigma(F^2^) is used only for calculating R-factors(gt) etc. and is not relevant to the choice of reflections for refinement. R-factors based on F^2^ are statistically about twice as large as those based on F, and R- factors based on ALL data will be even larger.

### Fractional atomic coordinates and isotropic or equivalent isotropic displacement parameters (Å^2^)

**Table:**

	*x*	*y*	*z*	*U*_iso_*/*U*_eq_	
S1	0.58145 (5)	0.71011 (3)	0.55769 (7)	0.02664 (12)	
F1	0.83575 (15)	0.34959 (9)	1.0261 (2)	0.0578 (4)	
O1	0.60897 (13)	0.48023 (8)	0.44611 (19)	0.0295 (3)	
O2	0.45182 (14)	0.73581 (10)	0.4371 (2)	0.0398 (4)	
C1	0.58675 (18)	0.61762 (12)	0.4704 (3)	0.0256 (4)	
C2	0.50850 (19)	0.59876 (12)	0.2895 (3)	0.0264 (4)	
C3	0.42931 (19)	0.64416 (13)	0.1364 (3)	0.0272 (4)	
H3	0.4163	0.7023	0.1383	0.033*	
C4	0.36966 (19)	0.60334 (14)	−0.0189 (3)	0.0312 (5)	
C5	0.3890 (2)	0.51695 (14)	−0.0187 (3)	0.0333 (5)	
H5	0.3476	0.4894	−0.1254	0.040*	
C6	0.4665 (2)	0.47071 (14)	0.1317 (3)	0.0318 (5)	
H6	0.4784	0.4122	0.1306	0.038*	
C7	0.52538 (18)	0.51362 (13)	0.2826 (3)	0.0280 (5)	
C8	0.64494 (19)	0.54532 (13)	0.5576 (3)	0.0276 (4)	
C9	0.2871 (2)	0.65312 (15)	−0.1847 (3)	0.0336 (5)	
H9	0.2812	0.7112	−0.1553	0.040*	
C10	0.1585 (2)	0.61773 (18)	−0.3167 (3)	0.0428 (6)	
H10A	0.1236	0.6148	−0.2651	0.051*	
H10B	0.1611	0.5601	−0.3488	0.051*	
C11	0.0782 (2)	0.6728 (2)	−0.4780 (3)	0.0504 (7)	
H11A	−0.0030	0.6471	−0.5636	0.061*	
H11B	0.0688	0.7288	−0.4477	0.061*	
C12	0.1316 (2)	0.68312 (19)	−0.5568 (3)	0.0516 (7)	
H12A	0.0810	0.7227	−0.6548	0.062*	
H12B	0.1305	0.6282	−0.6015	0.062*	
C13	0.2609 (2)	0.71598 (18)	−0.4262 (3)	0.0489 (7)	
H13A	0.2610	0.7741	−0.3931	0.059*	
H13B	0.2952	0.7172	−0.4788	0.059*	
C14	0.3392 (2)	0.66032 (16)	−0.2663 (3)	0.0399 (6)	
H14A	0.3452	0.6036	−0.2981	0.048*	
H14B	0.4218	0.6841	−0.1814	0.048*	
C15	0.73455 (18)	0.52553 (13)	0.7407 (3)	0.0284 (4)	
C16	0.8090 (2)	0.58784 (14)	0.8612 (3)	0.0367 (5)	
H16	0.8054	0.6430	0.8240	0.044*	
C17	0.8882 (2)	0.57015 (16)	1.0350 (3)	0.0449 (6)	
H17	0.9367	0.6138	1.1160	0.054*	
C18	0.8980 (2)	0.49001 (16)	1.0925 (3)	0.0430 (6)	
H18	0.9524	0.4775	1.2118	0.052*	
C19	0.8258 (2)	0.42899 (14)	0.9703 (3)	0.0388 (6)	
C20	0.7442 (2)	0.44338 (14)	0.7971 (3)	0.0335 (5)	
H20	0.6957	0.3992	0.7174	0.040*	
C21	0.6568 (2)	0.78020 (13)	0.5232 (3)	0.0344 (5)	
H21A	0.7411	0.7613	0.5918	0.041*	
H21B	0.6149	0.7796	0.4003	0.041*	
C22	0.6564 (3)	0.86840 (15)	0.5760 (4)	0.0450 (6)	
H22A	0.6982	0.8687	0.6977	0.067*	
H22B	0.5728	0.8873	0.5062	0.067*	
H22C	0.6981	0.9063	0.5581	0.067*	

### Atomic displacement parameters (Å^2^)

**Table:**

	*U*^11^	*U*^22^	*U*^33^	*U*^12^	*U*^13^	*U*^23^
S1	0.0314 (3)	0.0225 (2)	0.0276 (2)	−0.0004 (2)	0.0208 (2)	−0.0017 (2)
F1	0.0739 (11)	0.0365 (8)	0.0653 (10)	0.0167 (7)	0.0484 (10)	0.0247 (7)
O1	0.0322 (8)	0.0221 (7)	0.0342 (8)	−0.0004 (6)	0.0227 (7)	−0.0020 (6)
O2	0.0349 (9)	0.0331 (8)	0.0494 (10)	0.0030 (7)	0.0280 (9)	−0.0051 (7)
C1	0.0253 (10)	0.0232 (10)	0.0292 (10)	−0.0023 (8)	0.0190 (9)	−0.0019 (8)
C2	0.0274 (11)	0.0246 (10)	0.0329 (11)	−0.0038 (8)	0.0229 (10)	−0.0056 (9)
C3	0.0276 (11)	0.0244 (11)	0.0286 (11)	−0.0008 (8)	0.0188 (9)	−0.0029 (8)
C4	0.0282 (11)	0.0370 (12)	0.0308 (11)	−0.0022 (9)	0.0210 (10)	−0.0052 (9)
C5	0.0356 (13)	0.0347 (12)	0.0362 (13)	−0.0107 (10)	0.0271 (11)	−0.0141 (10)
C6	0.0369 (12)	0.0269 (11)	0.0434 (13)	−0.0050 (9)	0.0320 (11)	−0.0086 (10)
C7	0.0275 (11)	0.0254 (10)	0.0363 (12)	−0.0016 (8)	0.0238 (10)	−0.0012 (9)
C8	0.0268 (11)	0.0244 (10)	0.0339 (11)	−0.0031 (8)	0.0216 (10)	−0.0020 (9)
C9	0.0346 (12)	0.0344 (12)	0.0278 (11)	−0.0030 (9)	0.0197 (10)	−0.0071 (9)
C10	0.0324 (13)	0.0575 (16)	0.0352 (13)	−0.0044 (11)	0.0218 (11)	−0.0027 (11)
C11	0.0326 (13)	0.0697 (18)	0.0335 (15)	0.0013 (13)	0.0165 (12)	0.0023 (12)
C12	0.0475 (16)	0.0666 (18)	0.0285 (13)	−0.0011 (13)	0.0213 (13)	−0.0014 (13)
C13	0.0506 (16)	0.0627 (18)	0.0363 (14)	−0.0076 (13)	0.0308 (13)	−0.0033 (12)
C14	0.0368 (14)	0.0499 (14)	0.0327 (12)	−0.0079 (11)	0.0236 (11)	−0.0043 (11)
C15	0.0251 (11)	0.0269 (11)	0.0344 (12)	0.0033 (8)	0.0208 (10)	0.0044 (9)
C16	0.0297 (12)	0.0310 (12)	0.0370 (12)	−0.0006 (10)	0.0178 (11)	0.0060 (10)
C17	0.0337 (13)	0.0430 (14)	0.0369 (14)	−0.0021 (10)	0.0159 (12)	−0.0002 (11)
C18	0.0364 (14)	0.0501 (15)	0.0340 (13)	0.0107 (11)	0.0207 (12)	0.0121 (11)
C19	0.0394 (14)	0.0333 (13)	0.0502 (15)	0.0150 (10)	0.0331 (13)	0.0181 (11)
C20	0.0358 (13)	0.0259 (11)	0.0430 (13)	0.0042 (9)	0.0285 (12)	0.0030 (9)
C21	0.0406 (13)	0.0272 (11)	0.0447 (13)	−0.0064 (9)	0.0328 (12)	−0.0050 (10)
C22	0.0591 (17)	0.0266 (12)	0.0598 (16)	−0.0072 (11)	0.0447 (15)	−0.0078 (11)

### Geometric parameters (Å, º)

**Table:**

S1—O2	1.4920 (16)	C11—H11A	0.9900
S1—C1	1.772 (2)	C11—H11B	0.9900
S1—C21	1.804 (2)	C12—C13	1.524 (4)
F1—C19	1.361 (3)	C12—H12A	0.9900
O1—C8	1.377 (2)	C12—H12B	0.9900
O1—C7	1.381 (3)	C13—C14	1.527 (4)
C1—C8	1.359 (3)	C13—H13A	0.9900
C1—C2	1.445 (3)	C13—H13B	0.9900
C2—C7	1.391 (3)	C14—H14A	0.9900
C2—C3	1.396 (3)	C14—H14B	0.9900
C3—C4	1.390 (3)	C15—C16	1.388 (3)
C3—H3	0.9500	C15—C20	1.405 (3)
C4—C5	1.406 (3)	C16—C17	1.382 (3)
C4—C9	1.514 (3)	C16—H16	0.9500
C5—C6	1.385 (3)	C17—C18	1.379 (3)
C5—H5	0.9500	C17—H17	0.9500
C6—C7	1.373 (3)	C18—C19	1.375 (4)
C6—H6	0.9500	C18—H18	0.9500
C8—C15	1.459 (3)	C19—C20	1.367 (3)
C9—C14	1.526 (3)	C20—H20	0.9500
C9—C10	1.531 (3)	C21—C22	1.514 (3)
C9—H9	1.0000	C21—H21A	0.9900
C10—C11	1.531 (4)	C21—H21B	0.9900
C10—H10A	0.9900	C22—H22A	0.9800
C10—H10B	0.9900	C22—H22B	0.9800
C11—C12	1.519 (4)	C22—H22C	0.9800
			
O2—S1—C1	106.30 (10)	C11—C12—H12A	109.3
O2—S1—C21	107.36 (11)	C13—C12—H12A	109.3
C1—S1—C21	98.42 (10)	C11—C12—H12B	109.3
C8—O1—C7	106.23 (15)	C13—C12—H12B	109.3
C8—C1—C2	107.06 (18)	H12A—C12—H12B	107.9
C8—C1—S1	125.93 (17)	C12—C13—C14	110.7 (2)
C2—C1—S1	125.60 (16)	C12—C13—H13A	109.5
C7—C2—C3	119.02 (19)	C14—C13—H13A	109.5
C7—C2—C1	105.08 (18)	C12—C13—H13B	109.5
C3—C2—C1	135.88 (18)	C14—C13—H13B	109.5
C4—C3—C2	119.28 (19)	H13A—C13—H13B	108.1
C4—C3—H3	120.4	C9—C14—C13	111.6 (2)
C2—C3—H3	120.4	C9—C14—H14A	109.3
C3—C4—C5	119.4 (2)	C13—C14—H14A	109.3
C3—C4—C9	119.34 (19)	C9—C14—H14B	109.3
C5—C4—C9	121.28 (19)	C13—C14—H14B	109.3
C6—C5—C4	122.20 (19)	H14A—C14—H14B	108.0
C6—C5—H5	118.9	C16—C15—C20	119.3 (2)
C4—C5—H5	118.9	C16—C15—C8	120.53 (19)
C7—C6—C5	116.7 (2)	C20—C15—C8	120.2 (2)
C7—C6—H6	121.7	C17—C16—C15	120.4 (2)
C5—C6—H6	121.7	C17—C16—H16	119.8
C6—C7—O1	125.95 (18)	C15—C16—H16	119.8
C6—C7—C2	123.4 (2)	C18—C17—C16	121.0 (2)
O1—C7—C2	110.60 (17)	C18—C17—H17	119.5
C1—C8—O1	111.02 (19)	C16—C17—H17	119.5
C1—C8—C15	132.3 (2)	C19—C18—C17	117.3 (2)
O1—C8—C15	116.66 (18)	C19—C18—H18	121.3
C4—C9—C14	111.50 (19)	C17—C18—H18	121.3
C4—C9—C10	113.44 (19)	F1—C19—C20	118.3 (2)
C14—C9—C10	109.39 (18)	F1—C19—C18	117.6 (2)
C4—C9—H9	107.4	C20—C19—C18	124.1 (2)
C14—C9—H9	107.4	C19—C20—C15	117.8 (2)
C10—C9—H9	107.4	C19—C20—H20	121.1
C9—C10—C11	110.9 (2)	C15—C20—H20	121.1
C9—C10—H10A	109.5	C22—C21—S1	109.59 (16)
C11—C10—H10A	109.5	C22—C21—H21A	109.8
C9—C10—H10B	109.5	S1—C21—H21A	109.8
C11—C10—H10B	109.5	C22—C21—H21B	109.8
H10A—C10—H10B	108.0	S1—C21—H21B	109.8
C12—C11—C10	111.5 (2)	H21A—C21—H21B	108.2
C12—C11—H11A	109.3	C21—C22—H22A	109.5
C10—C11—H11A	109.3	C21—C22—H22B	109.5
C12—C11—H11B	109.3	H22A—C22—H22B	109.5
C10—C11—H11B	109.3	C21—C22—H22C	109.5
H11A—C11—H11B	108.0	H22A—C22—H22C	109.5
C11—C12—C13	111.7 (2)	H22B—C22—H22C	109.5
			
O2—S1—C1—C8	−131.38 (19)	C3—C4—C9—C14	111.2 (2)
C21—S1—C1—C8	117.7 (2)	C5—C4—C9—C14	−67.5 (3)
O2—S1—C1—C2	33.2 (2)	C3—C4—C9—C10	−124.7 (2)
C21—S1—C1—C2	−77.72 (19)	C5—C4—C9—C10	56.6 (3)
C8—C1—C2—C7	0.9 (2)	C4—C9—C10—C11	177.6 (2)
S1—C1—C2—C7	−166.14 (15)	C14—C9—C10—C11	−57.3 (3)
C8—C1—C2—C3	−177.8 (2)	C9—C10—C11—C12	56.1 (3)
S1—C1—C2—C3	15.2 (4)	C10—C11—C12—C13	−54.4 (3)
C7—C2—C3—C4	−0.2 (3)	C11—C12—C13—C14	54.2 (3)
C1—C2—C3—C4	178.4 (2)	C4—C9—C14—C13	−175.7 (2)
C2—C3—C4—C5	0.8 (3)	C10—C9—C14—C13	58.0 (3)
C2—C3—C4—C9	−177.90 (19)	C12—C13—C14—C9	−56.6 (3)
C3—C4—C5—C6	−0.4 (3)	C1—C8—C15—C16	−24.8 (4)
C9—C4—C5—C6	178.3 (2)	O1—C8—C15—C16	155.5 (2)
C4—C5—C6—C7	−0.8 (3)	C1—C8—C15—C20	153.6 (2)
C5—C6—C7—O1	−177.80 (19)	O1—C8—C15—C20	−26.1 (3)
C5—C6—C7—C2	1.5 (3)	C20—C15—C16—C17	−2.7 (3)
C8—O1—C7—C6	179.5 (2)	C8—C15—C16—C17	175.7 (2)
C8—O1—C7—C2	0.2 (2)	C15—C16—C17—C18	2.1 (4)
C3—C2—C7—C6	−1.0 (3)	C16—C17—C18—C19	−0.1 (4)
C1—C2—C7—C6	179.99 (19)	C17—C18—C19—F1	179.4 (2)
C3—C2—C7—O1	178.33 (18)	C17—C18—C19—C20	−1.2 (4)
C1—C2—C7—O1	−0.6 (2)	F1—C19—C20—C15	179.94 (19)
C2—C1—C8—O1	−0.8 (2)	C18—C19—C20—C15	0.6 (3)
S1—C1—C8—O1	166.15 (14)	C16—C15—C20—C19	1.4 (3)
C2—C1—C8—C15	179.5 (2)	C8—C15—C20—C19	−177.0 (2)
S1—C1—C8—C15	−13.5 (4)	O2—S1—C21—C22	65.6 (2)
C7—O1—C8—C1	0.4 (2)	C1—S1—C21—C22	175.71 (18)
C7—O1—C8—C15	−179.85 (17)		

### Hydrogen-bond geometry (Å, º)

**Table:**

*D*—H···*A*	*D*—H	H···*A*	*D*···*A*	*D*—H···*A*
C16—H16···O2^i^	0.95	2.54	3.291 (3)	136
C3—H3···F1^ii^	0.95	2.54	3.438 (3)	159

Symmetry codes: (i) *x*+1/2, −*y*+3/2, *z*+1/2; (ii) *x*−1/2, *y*+1/2, *z*−1.

## References

[bb1] Brandenburg, K. (1998). *DIAMOND* Crystal Impact GbR, Bonn, Germany.

[bb2] Bruker (2009). *APEX2*, *SADABS* and *SAINT* Bruker AXS Inc., Madison, Wisconsin, USA.

[bb3] Choi, H. D., Seo, P. J. & Lee, U. (2012). *Acta Cryst.* E**68**, o944. 10.1107/S1600536812008343 PMC3343925 22590006

[bb4] Choi, H. D., Seo, P. J., Son, B. W. & Lee, U. (2011). *Acta Cryst.* E**67**, o470. 10.1107/S1600536811002297 PMC3051523 21523129

[bb5] Farrugia, L. J. (2012). *J. Appl. Cryst.* **45**, 849–854.

[bb6] Flack, H. D. (1983). *Acta Cryst.* A**39**, 876–881.

[bb7] Sheldrick, G. M. (2008). *Acta Cryst.* A**64**, 112–122. 10.1107/S0108767307043930 18156677

